# Plasma transfusion in neonatal sepsis

**DOI:** 10.1007/s12519-026-01095-6

**Published:** 2026-08-28

**Authors:** Fang-Rui Ding, Xiao-Yan Li

**Affiliations:** 1 Department of Neonatology, Tianjin Central Hospital of Obstetrics and Gynecology, Tianjin, China; 2 Tianjin Key Laboratory of Human Development and Reproductive Regulation, Tianjin, China; 3 Department of Neonatology, Nankai University Maternity Hospital, Tianjin, China; 4 Neonatal Intensive Care Unit, Shanxi Province Children’s Hospital, Taiyuan, China

In neonates, sepsis is one of the leading causes of mortality globally [1–4]. Population meta-analyses and Global Burden of Disease (GBD) data reveal a huge disease burden: the worldwide incidence reaches 2824 per 100,000 live births with an overall mortality of 17.6%, and 206,451 neonatal sepsis-related deaths were recorded globally in 2021 [1, 3]. Rigional disparity occurs in nations with different social economic level. In high-income nations, such as Switzerland, neonatal intensive care unit (NICU) sepsis incidence (1.3%) and mortality (10%) are low. While in low-resource areas like Uganda, hospitalization sepsis rate is high (35.5%) [5–9].

Beyond the regional discrepancy, specific biological characteristics further complicate the management of neonatal sepsis. On the one hand, the neonatal immune system is not yet fully developed [10–13]. As a consequence, once sepsis occurs, neonates are prone to rapid progression to septic shock and multiple organ dysfunction syndrome (MODS) [10–13]. On the other hand, the metabolic and enzyme systems of the liver and kidneys in neonates are not yet fully developed, and their ability to clear drugs is weak. Antibiotics, such as fluoroquinolones and sulfonamides, commonly used in adult clinical practice cannot be directly used in neonates [14–20].

Collectively, the current management of neonatal sepsis presents a significant challenge. Against this backdrop, research has increasingly focused on adjunctive therapies built upon standard antibiotic treatment, including plasma transfusion [11, 16–20]. However, timing and precise indications for plasma transfusion in neonates are controversial [16, 18, 20–22]. This editorial discusses theoretical foundation for treatment, risks, current clinical application, and relevant research. We aim to provide reference material and support for subsequent research and rational clinical use.

## Theoretical foundations and potential benefits of plasma transfusion in the treatment of sepsis

The rationale for attempting plasma transfusion as a treatment for sepsis originated from use in trauma patients [23–27]. This was also inferred from the high degree of pathophysiological similarity between trauma and sepsis [28, 29]. First, both conditions trigger acute intravascular volume depletion and tissue hypoperfusion: systemic vasodilation and elevated vascular permeability drive massive fluid extravasation, which destabilizes the circulation leading to a rapidly progressing to shock. Second, both disorders induce severe coagulopathy, marked by excessive consumption of coagulation factors, disrupted anticoagulant pathways, and dysregulated fibrinolysis. Given these overlapping pathogenic mechanisms and multiple studies demonstrating early and active plasma transfusion in trauma can significantly improve both survival and clinical outcomes, it is biologically plausible to infer that plasma transfusion, effective in trauma, may confer comparable therapeutic benefits for patients with sepsis [23–30].

Neonates, especially preterm infants, have inherently low levels of coagulation factors, insufficient immunoglobulins and complement, and a more fragile vascular endothelial barrier. When sepsis occurs, the systemic inflammatory response triggered by infection will further consume immune and coagulation-related mediators, leading to low immune function, coagulopathy, and worsening of disease progression [31–33]. A further potential benefit of plasma transfusion lies in the rapid supplementation of deficient bioactive substances. Theoretically, immunoglobulin replenishment could strengthen specific immune defense against pathogens, neutralize pathogenic toxins, and facilitate infection control. It could also supplement coagulation factors and fibronectin to optimize coagulation, thereby preventing and treating sepsis-associated coagulopathy. In addition, plasma also provides complement components to boost bacteriolytic and opsonizing capacity and promote pathogen clearance. Moreover, colloid supplementation may help maintain both blood volume and plasma osmotic pressure to improve tissue perfusion and correct septic shock [34–39].

Animal studies have indeed confirmed the above theoretical inferences [40–42]. Chang et al. established a septic rat model and found that plasma transfusion raised the 48-hour survival rate from 14% to 57% relative to normal saline [40]. McTaggart et al. demonstrated that plasma transfusion elevated serum immunoglobulin G (IgG) levels in both healthy and septic foals; although it exerted no beneficial effect on neutrophil function in healthy foals, it significantly improved neutrophil activity in septic foals, further validating the therapeutic benefits of plasma transfusion for sepsis [41]. Pereira et al. used neonatal dogs with sepsis, combined treatment with plasma and antibiotics improved immune and metabolic parameters including blood glucose, white blood cell counts and IgM levels within 24 hours, shortened the recovery time of leukocyte indices, reduced lactate levels, and resulted in zero mortality. For comparison, there was a 22% mortality rate in the antibiotic-only group [42].

Despite evidences from theoretical and animal studies, no high-grade human evidence has confirmed the clinical efficacy of plasma transfusion for neonatal sepsis. In the absence of this high-quality human data, the potential benefits demonstrated in animal models should be interpreted with caution because they may not be directly applicable to human neonates due to the inherent differences in species and environment.

### Clinical evidence and safety risks of plasma transfusion in the treatment of sepsis

Benefits of plasma transfusion in the treatment of sepsis are still conflicting. Moreover, transfusion was found to be associated with increased incidence and poor prognosis [43–60].

Qin et al. performed a cohort study utilizing data extracted from the Medical Information Mart for Intensive Care III database. This study indicated that in the plasma transfusion group, both 28-day and 90-day mortality were significantly higher than in the non-transfusion group. In addition, plasma transfusion did not improve the prognosis of sepsis patients with coagulopathy, regardless of whether they were in a hypocoagulatory state or not. External validation results were consistent with the conclusions of the main study [43].

In a pediatric sepsis study conducted by Duan et al., a total of 262 children with sepsis were recruited. Patients were categorized into two groups based on whether they received plasma transfusion. As a result, 28-day mortality, in-hospital mortality, and MODS morbidity were significantly higher in the plasma transfusion group than in the non-plasma group. Plasma transfusion did not demonstrate benefit and was instead significantly associated with an increased risk of adverse outcomes [44].

In addition to clinical efficacy, several studies have reported severe adverse consequences of plasma transfusion. Indeed, plasma transfusion is associated with allergic reactions, transfusion-related acute lung injury (TRALI), transfusion-associated circulatory overload (TACO), and the transmission of bacterial or viral infections. Allergic reactions may occur at an approximate incidence of 1%–3% [45]. As for TRALI, better mechanistic understanding and optimized collection has significantly reduced the risks. However, the incidence of TRALI in critically ill patients is estimated to be 0.08%–15.1%, and the proportion of transfusion-related fatalities is 34% [46–49]. Another adverse reaction of plasma transfusion is TACO, with an incidence of about 1%. In critically ill patients, this can reach 3.6%–5.8%, and there are widespread cases of missed diagnosis and underreporting [50–56]. With respect to transfusion-related infections, while the risk has been substantially reduced through donor screening, testing, and isolation measures, residual risks remain. Transmission rates for human immunodeficiency virus (HIV) and hepatitis C virus (HCV) are approximately 0.02 per 10,000 transfusions. However, newly emerging pathogens may also present potential threats [57, 58]. In addition, plasma transfusion may increase the risk of nosocomial infections via transfusion-related immunomodulation; this is associated with postoperative infection in critically ill patients [59–62].

### Current status of plasma utilization in neonatal sepsis

Plasma transfusion is relatively common in the NICU; its use varies significantly across different centers from 0.17% to 8.2% [22, 23, 63–69]. Generally, the overall characteristic is that plasma transfusion rates are higher for surgical cases and extremely preterm neonates; indications for plasma transfusion vary among different regions [22, 23, 63–69].

The clinical situations in which plasma is used for neonates mainly include surgical procedures, congenital coagulation disorders, bleeding, disseminated intravascular coagulation (DIC), prophylactic use including sepsis and others [22, 23, 63–69]. There is little controversy regarding indications for plasma use in surgical cases and bleeding. However, significant controversy remains in the prophylactic use of plasma and its use in other clinical situations including sepsis.

We have summarized recent (2014–2026) plasma transfusion practice in Table 1 [22, 65, 69–79]. It is clear that these studies do not focus on the efficacy of plasma transfusion in neonatal sepsis. Most aim to clarify the epidemiology and indications for plasma transfusion. There is a long-standing lack of high-quality human randomized controlled trial (RCT) studies in this field. Indeed, few articles have focused on the impact of plasma transfusion on neonatal sepsis. A study conducted by Acunas et al. in 1994 involving 67 infected neonates and 30 healthy controls found that while intravenous immunoglobulin (IVIG) exerted prominent immune-enhancing and anti-inflammatory effects, plasma transfusion failed to elevate key anti-infective IgG or alter inflammatory markers including C-reactive protein and fibronectin; this indicates no clinical benefits for neonatal sepsis [19]. A Nigerian retrospective study by Ogunlesi TA et al., involving 49 neonates with tetanus complicated by suspected sepsis, observed a lower mortality rate in seven plasma-transfused critically ill neonates versus 42 untreated controls though the difference was statistically insignificant due to the small sample size. Of note, all blood culture-positive patients in the transfusion group survived, suggesting screened fresh plasma may be a promising adjuvant immunotherapy in resource-limited high-burden regions, pending large-scale randomized verification [80]. In addition, Eisenfeld et al. demonstrated that plasma transfusion significantly improved neutrophil chemotactic function in critically ill neonates with suspected severe sepsis, particularly in confirmed sepsis cases, indicating plasma can boost neonatal innate immune defense by enhancing neutrophil migration to infection sites [81].

**Table 1:** Clinical practice for plasma transfusion in neonates

Author	Publication year	Country	Study type and design	Intervention	Objectives	Results	Neonatal sepsis outcomes involved
Motta et al. [69]	2014	Italy	Prospective observational study	Plasma transfusion	To quantify the compliance of plasma transfusion with guideline recommendations	60% of plasma transfusions were non-compliant with published guidelines	No
Raban et al. [70]	2015	South Africa	Retrospective observational study	Plasma transfusion	To examine indications and compliance of plasma transfusion with published guidelines	75% of plasma transfusions complied with national guidelines	No
Priya R et al. [71]	2018	India	Cross-sectional observational study	Plasma transfusion	To estimate outcomes of plasma transfusion in preterm neonates	66.7% of infants showed a decrease and 33.3% infants showed an increase from pretransfusion PT and INR values	No
Dogra et al. [72]	2020	India	Prospective cohort study	Plasma transfusion	To examine relationship of plasma transfusion on clinical outcome of neonates admitted in NICU	Transfusion of plasma does not confer benefits for clinical bleeding, but it significantly alters laboratory parameters	No
Sharif et al. [73]	2020	India	Retrospective observational study	Plasma transfusion	To evaluate rational use of the plasma	71% of plasma transfusions were appropriate	No
Houben et al. [74]	2020	Netherlands	Retrospective cohort study	Plasma transfusion	To evaluate changes in the use of plasma transfusions and the use of clotting tests in preterm neonates over the past two decades	Most transfusions were administered prophylactically based on the prolongation of aPTT or PT, DIC (20.2% of cases) or other causes of abnormal coagulation (56.3% of cases)	No
Amrutiya et al. [65]	2020	India	Retrospective cohort study	Plasma transfusion	To audit plasma transfusion indications	The most frequent indication was neonatal sepsis along with significant changes in coagulation parameters after transfusion	No
Patel et al. [75]	2021	USA	Retrospective cohort study	Plasma transfusion	To estimate the incidence of plasma transfusion and characterize pretransfusion hematology values	The incidence of plasma transfusion was 0.7%. Median pretransfusion international normalized ratio was 1.7	No
Scrivens et al. [76]	2023	18 European countries	Cross-sectional observational study	Plasma transfusion	To describe current transfusion practices in Europe	Plasma was used for coagulopathy with active bleeding in 93% NICUs; active bleeding without coagulopathy in 46% NICUs; coagulopathy without active bleeding in 39% NICUs; sepsis in 26.5% NICUs; and hypotension in 25% NICUs	No
Aziz et al. [77]	2024	USA	Retrospective cohort study	Plasma or cryoprecipitate transfusion	To measure the frequency of intervention with plasma for sepsis	Intervention (plasma or cryoprecipitate) followed in a minority (31%) of abnormal coagulation cases	No
Houben et al. [22]	2025	22 European countries	Prospective observational study	Plasma transfusion	To describe neonatal plasma transfusion practice in Europe	The primary indications for plasma transfusions were active bleeding (29.4%), abnormal coagulation screen results (23.7%) or volume replacement/hypotension (21.5%)	No
Aslan et al. [78]	2026	Turkey	Retrospective observational study	Plasma transfusion	To examine indications for plasma use in a neonatal inpatient population	Only 20.3% of transfusions met guidelines. Sepsis was one of indications for support treatment	No
Arora et al. [79]	2026	India	Prospective, cross-sectional study	Plasma transfusion	To characterize the indications for plasma transfusions among neonatal patients across India	The main indication for plasma transfusion was deranged coagulation	No

*PT* prothrombin time, *INR* international normalized ratio, *NICU* neonatal intensive care unit, *aPTT* activated partial thromboplastin time, *DIC* disseminated intravascular coagulation

Whilst studies on plasma transfusion in neonatal sepsis are limited, there are data on the use of neonatal plasma transfusion in other clinical scenarios [82–90]. These findings require careful consideration when administering plasma for the treatment of sepsis. They offer valuable insights into other clinical outcomes. Several studies have reported that plasma transfusion is associated with retinopathy of prematurity (ROP), bronchopulmonary dysplasia (BPD), hemodynamically significant patent ductus arteriosus (hsPDA), and necrotizing enterocolitis (NEC) [82–90].

Regarding ROP, data are controversial. Hadi et al. identified plasma transfusion as a risk factor for ROP in preterm infants [82]. In contrast, Dani et al. found that plasma transfusions within the first week after birth significantly reduced the risk of ROP [83]. Such contradictory findings stem from fundamental differences in study design and clinical context. This study, demonstrating a protective effect, exclusively enrolled extremely preterm infants less than 29 weeks’ gestation, with a separate analysis focusing on prophylactic plasma administered within the first postnatal week; multivariate adjustment confirmed that only early, repeated prophylactic plasma transfusions could lower ROP risk. By comparison, the study linking plasma transfusion to elevated ROP risk included preterm infants across a broader gestational window (25–35 weeks), where plasma transfusions were predominantly administered as emergency support for critically ill neonates instead of prophylactic use. This study also jointly analyzed red blood cell and plasma transfusions, accounting for the additive effects of RBC transfusion, ultimately concluding that plasma transfusion correlated with higher ROP risk. These discrepancies suggest that transfusion timing and clinical setting may have different effects on complications.

Equally conflicting results exist in NEC. Whilst Parvizian et al. suggested that plasma transfusion is associated with an increased incidence of NEC, while Osborn et al. studied early volume expansion using plasma and reported a reduced risk of NEC [84–86]. The former study involved neonates who had received at least one red blood cell (RBC) transfusion; within this group, plasma transfusion was found to elevate NEC risk. The main difference between the two studies lies in the characteristics of the population included. This naturally questions if the inconsistency is related to the application of the two blood products. It is plausible that residual RBC-derived antigens persisting in the circulation may interact with donor-derived antibodies present in plasma, thereby promoting RBC aggregation, increasing whole-blood viscosity, impairing intestinal microcirculatory perfusion, and ultimately contributing to NEC pathogenesis. These considerations caution the administration of multiple blood products, and their respective compositions, when evaluating the independent effects of plasma transfusion on NEC risk.

As for hsPDA, one study showed that early plasma transfusion could increase circulating blood volume, leading to an increased risk of developing hsPDA [87]. In addition to this early complication, in the long term, some studies reported that plasma transfusion is also a risk factor for the development of BPD [88]. In addition, prophylactic plasma transfusion was initially intended to prevent intraventricular hemorrhage. Several studies in this area have been performed over the last century [23, 89, 90]. Despite this, there is no clear confirmation that early prophylactic plasma transfusion could prevent intraventricular hemorrhage [89].

Collectively, factors, including transfusion timing, indication, administration of other blood products, and characteristics of the study population, can all directly influence the risk of various complications. This indicates that plasma transfusion requires attention to multiple potential adverse outcomes, rather than focusing solely on a single primary disease.

In summary, there are no definitive evidence-based medical data to confirm that plasma transfusion is beneficial in sepsis. However, this does not mean that there are no benefits; further studies and evidence are needed. We suggest that more retrospective and prospective large-sample studies are performed to provide reliable evidence-based medical advice for the application of plasma transfusion in neonatal sepsis.

### Current guidelines and recommendations for plasma transfusion in the treatment of neonatal sepsis

Current international guidelines in neonatal sepsis are consistent in their recommendations for the use of plasma, with the core principle being only for bleeding combined with coagulation disorders; prophylactic transfusion is not recommended. No recommendations for the use of plasma are found in current sepsis guidelines [91–93]. Although these guidelines do not provide recommendations [91–93], discussion can be found along with expert consensus on blood product transfusion [94–98]. We have summarized some country-specific and international guidelines in Table 2 [94–98].

**Table 2:** Plasma transfusion guidelines

Institution	Country	Recommendations or opinions on plasma transfusion in sepsis
British Committee for Standards in Hematology [94]	UK	Prophylactic use of plasma not recommended for sepsis without bleeding and without disseminated intravascular coagulation
Egyptian Pediatric Clinical Practice Guidelines Committee [95]	Egypt	Use for anti-infective purposes is strictly prohibited
Transfusion and Anemia Expertise Initiative-Control/Avoidance of Bleeding [96]	International	Recommended in patients with minimal, moderate or severe bleeding with sepsis and/or disseminated intravascular coagulation
Italian Society of Transfusion Medicine and Immunohematology and Italian Society of Neonatology working group [97]	Italy	The use of this blood component in sepsis or as a volume expander in the neonate with hypotension is not considered appropriate
National Health Commission of the People’s Republic of China [98]	China	Not mentioned

Though the recommendations for plasma transfusion in neonatal sepsis can be summarized as: plasma should be given to neonates with active bleeding and coagulopathy (Table 1), observational surveys consistently reveal widespread guideline non-adherence. Two core interrelated reasons underpin this persistent practice-guideline deviation: The main reason could be the lack of effective targeted interventions for neonatal septic shock. Neonatal sepsis progresses rapidly to irreversible MODS, and standard antibiotic, fluid, and vasoactive support often fails to stabilize circulatory collapse. Faced with deteriorating neonates with no proven rescue therapies, clinicians adopt plasma as a salvage adjunct out of clinical urgency, prioritizing perceived potential benefit over guideline-mandated restrictive thresholds. Second, the extrapolation of clinical experience in trauma resuscitation, along with the immature immune and coagulation systems in neonates, influences clinician decision-making. Neonatologists intuitively assume plasma will mitigate septic shock and inflammatory damage, and that plasma could supplement immune factors. This reasoning dominates empirical practice. In addition, adherence to guidelines does not correlate with adequacy of medical resources or whether a country is classified as high-income. As shown in Table 1, in European countries, only 29.4% of blood transfusions were for bleeding. In South Africa, 75% of plasma transfusions complied with national guidelines [22, 70]. Among the non-guideline compliant plasma transfusions, the transfusion indications vary. However, the core reason behind this is a lack of high-quality evidence resulting in region-specific adherence.

## Future research directions

### Multi-center and large-sample randomized controlled trial studies are needed

Although current guidelines have reached a high-level consensus on the indications for using plasma in patients with sepsis, the evidence supporting these indications is limited. It is mandatory to clarify clinical indications based on different situations encountered in neonatal sepsis. Plasma transfusion is not unfamiliar in the neonatal population but not all neonatal sepsis cases strictly adhere to indications for plasma transfusion. Based on previous data, researchers should initially conduct a retrospective analysis of the efficacy of plasma transfusion in the treatment of sepsis. With results of such a retrospective study, a large-sample, multicenter prospective RCT should be performed. Studies should carefully define inclusion and exclusion criteria and select appropriate outcome variables, including coagulation and infection. Optimal study population and timing need to be defined. Stratified analysis should be conducted on neonates of different gestational ages and weights to define differences in efficacy and provide a basis for individualized treatment.

### Mechanistic research is needed

At present, mechanistic data on plasma transfusion in the treatment of neonatal sepsis are superficial. In-depth exploration of the role of various bioactive substances in plasma (such as immunoglobulins, coagulation factors, complements, and fibronectin) should be made in neonatal sepsis.

### Safety studies need to consider both short-term and long-term adverse reactions

There should be an emphasis on TACO, virus transmission, and allergic reactions within the neonatal population. To achieve this, collaboration with hematologists to formulate an effective plasma processing plan and further mitigate the incidence of transfusion-related infectious and immune diseases is needed. In addition, for neonates, there are several special presentations, including BPD, ROP, and other systemic diseases that require a critical focus.

## Conclusions

International recommendations regarding plasma transfusion in neonatal sepsis are relatively consistent. None of these recommendations advocate for the routine use of plasma. This is only recommended for cases where bleeding is accompanied by coagulation abnormalities. However, the evidence supporting this recommendation is extremely limited. In addition, considering that the immune system of neonates, particularly preterm infants, is not fully developed, plasma may theoretically compensate for this deficiency. Further studies on the use and timing of plasma transfusion in neonatal sepsis are essential.

## Data Availability

Not applicable.

## Declarations

### AI use statement

No artificial intelligence was used in this work.
